# The Bluetongue Disabled Infectious Single Animal (DISA) Vaccine Platform Based on Deletion NS3/NS3a Protein Is Safe and Protective in Cattle and Enables DIVA

**DOI:** 10.3390/v13050857

**Published:** 2021-05-07

**Authors:** Piet A. van Rijn, Mieke A. Maris-Veldhuis, René G. P. van Gennip

**Affiliations:** 1 Department of Virology, Wageningen Bioveterinary Research (WBVR), 8200 Lelystad, The Netherlands; mieke.maris@wur.nl (M.A.M.-V.); rene.vangennip@wur.nl (R.G.P.v.G.); 2 Department of Biochemistry, Centre for Human Metabolomics, North-West University, Potchefstroom 2520, South Africa

**Keywords:** bluetongue virus, vaccine platform, DISA principle, DIVA principle, NS3/NS3a protein, reverse genetics, cattle

## Abstract

The bluetongue virus (BTV) is transmitted by *Culicoides* biting midges and causes bluetongue (BT), an OIE-notifiable disease of ruminants. At least 29 BTV serotypes are described as determined by the outer shell proteins VP2 and VP5. Vaccination is the most effective control measure. Inactivated and live-attenuated vaccines (LAVs) are currently available. These vaccines have their specific pros and cons, and both are not DIVA vaccines. The BT Disabled Infectious Single Animal (DISA) vaccine platform is based on LAV without nonessential NS3/NS3a expression and is applicable for many serotypes by the exchange of outer shell proteins. The DISA vaccine is effective and completely safe. Further, transmission of the DISA vaccine by midges is blocked (DISA principle). Finally, the DISA vaccine enables DIVA because of a lack of antibodies against the immunogenic NS3/NS3a protein (DIVA principle). The deletion of 72 amino acids (72aa) in NS3/NS3a is sufficient to block virus propagation in midges. Here, we show that a prototype DISA vaccine based on LAV with the 72aa deletion enables DIVA, is completely safe and induces a long-lasting serotype-specific protection in cattle. In conclusion, the in-frame deletion of 72-aa codons in the BT DISA/DIVA vaccine platform is sufficient to fulfil all the criteria for modern veterinary vaccines.

## 1. Introduction

Bluetongue (BT) is a severe disease of ruminants caused by the bluetongue virus (BTV) and characterized by fever, oral and nasal erosions and discharges, edema, coronitis, anorexia and death caused by damage of the vascular endothelium [1,2]. Generally, sheep are more susceptible to BT than cattle and goats. BT, together with African Horse Sickness and Enzootic Hemorrhagic Disease, are notifiable midge-borne animal diseases [3]. Today, more than 29 BTV serotypes have been recognized based on serum neutralizing tests (SNTs), protection studies and phylogenetic analyses [4,5,6,7,8,9,10]. Serotypes 1-24 are transmitted by bites of *Culicoides* midges [11]. In contrast, BTV serotypes 25-27 are not pathogenic, cannot be propagated in midge cells and are spread by in-contact transmission [12,13,14]. Recently discovered serotypes 28 and 29 are still less-studied [8,15], and the number of serotypes is still being counted [16,17]. Outbreaks of BTV1-24 can lead to large economic damage by diseased sheep and trade losses and movement restrictions for all kind of ruminants, including cattle [18,19]. Cattle are less susceptible to BT but are considered as a reservoir host and a major source for the onward spread of the disease by midges [20,21].

BTV is the prototype orbivirus of the genus *Orbivirus* of the family of *Reoviridae* [22]. Orbivirus is a triple-layered nonenveloped virus containing a ten-segmented genome of double-stranded RNA (Seg-1–10). The genome encodes seven structural virus proteins VP1-7 and at least four nonstructural proteins: NS1-4 [22,23,24,25]. The outer shell proteins are encoded by Seg-2[VP2] and Seg-6[VP5], of which VP2 is the major serotype specific immunodominant protein to raise neutralizing antibodies (nAbs) [26]. Many BTV serotypes are endemic in large parts of the tropical world related to the presence of competent *Culicoides* vector species [27]. BTV serotypes 2, 10, 11, 13 and 17 are endemic in the USA [28]. Re-emerging outbreaks and incidents of BTV serotypes 1, 2, 3, 4, 6, 8, 9, 11, 14 and 16 have been frequently reported in the European Union in the last two decades.

Currently, inactivated vaccines and live-attenuated vaccines (LAVs) are used in different parts of the world depending on the local situation and their specific pros and cons [29]. Both vaccine types do not enable the differentiation of infected from vaccinated animals (DIVA principle) [30]. The research has resulted in new BT vaccine candidates, like protein vaccines, viral vector vaccines and modified-live BT vaccine platforms, such as Disabled Infectious Single-Cell Cycle (DISC) vaccine and Disabled Infectious Single Animal (DISA) vaccine, reviewed in [31]. The key of the experimental BT Disabled Infectious Single Animal (DISA) vaccine platform is the deletion of the NS3/NS3a protein, which is not essential for virus growth in vitro [32]. NS3/NS3a encoded by Seg-10 is a multifunctional membrane-associated protein playing a crucial role in virus propagation in blood-fed midges (DISA principle) [33], in virus release [32,34], in the suppression of the cellular interferon response through different mechanisms [35,36,37], in virulence [38] and is immunogenic [39,40]. The lack of functional NS3/NS3a results in a safe, protective, cost-competitive, nontransmittable and DIVA-compatible DISA vaccine [31]. Further, this DISA/DIVA vaccine platform is applicable for different serotypes by the exchange of the serotype immunodominant VP2 protein [41]. Recently, we showed that 72 amino acids (aa) encompassing the immunogenic Late Domain is essential for propagation in blood-fed midges [42]. The deletion of a similar region in Seg-10[NS3/NS3a] of the virulent African horse sickness virus, a related orbivirus species, resulted in a safe and protective AHS DISA/DIVA vaccine for ponies [43,44].

Here, we developed a prototype DISA/DIVA vaccine, named DISA8, based on LAV with a 72-aa deletion in Seg-10[NS3/NS3a] and the exchange of Seg-2[VP2] and Seg-6[VP6] of serotype 8. DISA8 meets all the vaccine criteria in cattle for modern veterinary vaccines, such as safety, efficacy, DISA and DIVA.

## 2. Materials and Methods

BSR cells, a clone of BHK-21 cells [45], were cultured in Dulbecco’s modified Eagle’s medium (DMEM, Fischer Scientific, Landsmeer, The Netherlands), containing 5% fetal bovine serum (FBS), 100-IU/mL Penicillin, 100-μg/mL Streptomycin and 2.5-µg/mL Amphotericin B (Fischer Scientific, Landsmeer, Netherlands).

BTV8/net07/e1/bhkp3 (BTV8) and BTV2/SAD01/01/e1/bhkp2/kcp3 (BTV2) have been described [46,47]. BTV8 was used as challenge virus, while BTV2 and BTV8 were used to determine the serotype-specific neutralizing antibodies (nAbs) in cattle sera.

**Figure 1:**
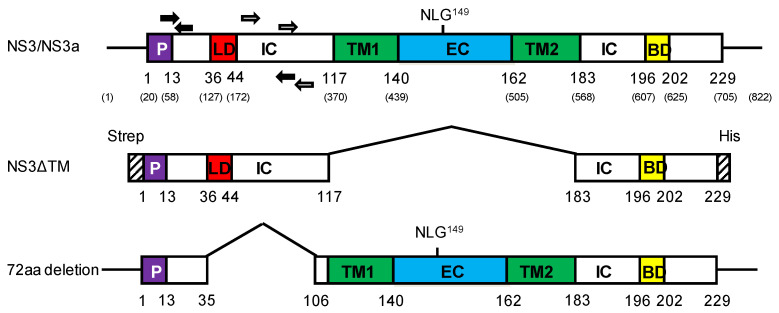
Schematic representation of Seg-10 and encoded NS3/NS3a proteins. Putative protein expression is indicated by boxes, whereas untranslated sequences are shown by lines. Calpactin p11-binding domain (P), Late Domain (LD), two transmembrane regions (TM1 and TM2) with a conserved glycosylation sites in-between (NLG149) and the VP2-binding domain (BD) are indicated. The topological orientation is indicated by intracellular (IC) and extracellular (EC). Primers and probes of Seg-10 panBTV PCR tests are indicated by closed and open arrows for van Rijn et al. and the OIE-recommended assay, respectively. Numbers indicate the amino acid position, and numbers between parentheses indicate the nucleotide position. Seg-10[NS3/NS3a] is the full-length Seg-10 present in wild-type BTVs. NS3ΔTM is used as antigen in the experimental NS3 cELISA. 72-aa deletion is the deletion of Seg-10[NS3/NS3a] present in the DISA/DIVA vaccine platform.

LAV-related strain BTV6/net08 [48,49] with an in-frame 72-aa deletion in Seg-10[NS3/NS3a] and exchange of Seg-2[VP2] and Seg-6[VP5] of BTV8/net06 [50] was generated using reverse genetics, as previously described [46,51]. All cDNAs used for reverse genetics have been previously described [46], except for Seg10 of BTV6/net08 with the 72-aa deletion. This cDNA was synthesized by Genscript Corporation, Piscataway, NJ, USA in an appropriate plasmid enabling T7-driven RNA run-off transcription. The 72-aa deletion in Seg-10[NS3/NS3a] corresponds to nucleotide positions 124–339 encoding the aa positions 35–106 and encompasses the Late Domain motif PPXY/PTAP, the most immunogenic part of NS3/NS3a [39,42] (Figure 1).

Briefly, the virus was rescued by transfection plasmids expression BTV proteins followed by the transfection of ten run-off RNA transcripts of the foreseen genome constellation. The incorporation of the genome segments of interest were confirmed according to the standard procedures, namely for Seg-2[VP2] and Seg-6[VP5], by partial sequencing and by sequencing the entire open reading frame of Seg-10[NS3/NS3a] to confirm the 72-aa deletion. The rescued virus, designated DISA8, was grown by the infection of fresh BSR cells at a low multiplicity of infection (MOI). When >50% of cells in a duplicate well were immunostained using the anti-VP7 monoclonal antibody (MAb) ATCC-CRL-1875, DISA8 was harvested by freeze–thawing and centrifugation. The virus titer of DISA8 was determined by the endpoint dilution on BSR cells and expressed as TCID_50_/mL.

The vaccination challenge trial in cattle was performed under the guidelines of the European Community and was approved by the Committee on the Ethics of Animal Experiments of Wageningen Bioveterinary Research (permit number 2017.D-0070.002). Twelve Holstein Frisian cows of 6–10 months old, free of BTV and BTV antibodies (Abs), were obtained from a Dutch farm. Each group of four cattle were housed in separate pens and arrived one week before their respective start in order to synchronize the BTV8 challenge on the same day. Vaccinations were carried out intramuscularly (im) in the neck with 2 × 1 mL of 10^5^ TCID_50_/mL of DISA8. Group ‘Prime-boost’ was vaccinated twice with an interval of three weeks on day 0 (0 dpv) and 21 dpv, corresponding to 0 days post-boost vaccination (dpb). Group ‘Single’ was vaccinated once on the day, corresponding to 63 dpv/42 dpb of group ‘Prime-boost’.

The BTV8 challenge was carried out by subcutaneous (sc) infection in the neck at both sides with 4 × 1 mL 10^5^ TCID_50_/mL virulent BTV8 on the same day for all three groups. Thus, the day of the challenge of group ‘Control’ (0 dpc) corresponded to 84 dpv/63 dpb of group ‘Prime-boost’ and 21 dpv of group ‘Single’. All cattle were sacrificed three weeks later, corresponding to 105 dpv for group ‘Prime-boost’, 42 dpv for group ‘Single’ and 21 dpc for group ‘Control’. Body temperature and clinical signs were monitored throughout the experiment. Clinical signs were scored according to the clinical score table for bluetongue [38,47,52,53] (Supplementary Table S1). EDTA blood and serum were collected at the indicated days of the experiment.

EDTA blood samples were examined for BTV RNA with the panBTV PCR tests targeting Seg-1 [54] or Seg-10 [55]. The crossing point (Cp) values were calculated, and samples without the Cp value showing an increase of the OD640/530 were interpreted as 40, and negative samples were set at 45.

Sera were tested for VP7 antibodies Abs with the BT VP7 competition enzyme linked immunosorbent assay (VP7 cELISA), according to the supplier’s instructions (ID.Vet). The percentage of blocking was displayed as 100 minus, and the seroconversion cut-off was set at 50%, according to the supplier.

Sera were tested for NS3 Abs with the experimental NS3 competition ELISA (NS3 cELISA) [39]. Briefly, optimal dilutions of the coated NS3ΔTM antigen (Figure 1), mouse monoclonal antibody (MAb) 33H7 and conjugated rabbit anti-mouse Abs were used. One hundred-microliter twofold diluted serum samples in dilution buffer were tested. The OD450 was measured, and the percentage of blocking was calculated and displayed as 100 minus [39].

Sera were tested for neutralizing Abs (nAbs) with the serum neutralizing test (SNT) for serotype 2 and 8, as described [52,56]. Twofold dilutions of the serum samples were tested for the indicated days, namely days 0, 21, 84 and 105 for group ‘Prime-boost’; days 0, 21 and 42 for group ‘Single’ and days 0 and 21 for group ‘Control’.

## 3. Results

### 3.1. Rescue of DISA8

The LAV-related strain BTV6/net08 with the in-frame 72-aa deletion in Seg-10[NS3/NS3a] (Figure 1) and exchanged outer shell proteins of serotype 8 were successfully rescued and named DISA8. The incorporation of Seg-2[VP2] and Seg-6[VP5] of BTV8/net06 and the in-frame 72-aa deletion were confirmed by sequencing (not shown). As expected, prototype DISA8 was detected by the Seg-1 panBTV PCR test, whereas the Seg-10 panBTV PCR test failed (not shown). A virus stock contained 10^7^ TCID_50_/mL of DISA8 and was used to prepare the vaccine of the appropriate titer.

### 3.2. Vaccination Challenge Experiment in Cattle

DISA8 was studied in groups of four cattle on early (group ‘Single’) and lasting protection (group ‘Prime-boost’). The intramuscular vaccination of groups ‘Prime-boost’ and ‘Single’ was performed in such a time schedule to synchronize the BTV8 challenge for both the vaccination groups and the control group (group ‘Control’) on the same day (0 dpc). Lasting protection was studied 84 days after the prime vaccination, corresponding to 63 days post the boost vaccination (84 dpv/63 dpb) (group ‘Prime-boost’). Early protection was studied at three weeks after a single DISA8 vaccination (21 dpv) (group ‘Single’). Thus, group ‘Control’ served as challenge group for both vaccinated groups.

#### 3.2.1. DISA8 Is Safe and Protects against Bluetongue

**Figure 2:**
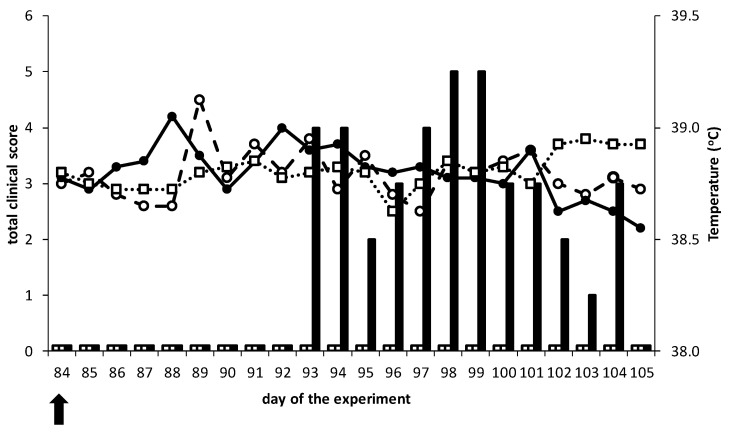
Body temperature and clinical signs after the challenge with virulent BTV8 in vaccinated and unvaccinated cattle. Group ‘Prime-boost’ received two vaccinations with an interval of three weeks on days 0 and 21 of the experiment. Group ‘Single’ received one DISA8 vaccination on day 63. No rise of body temperature nor clinical signs were observed after the DISA8 vaccination(s) up to 84 days (not shown). Both vaccinated groups, together with the unvaccinated group ‘Control’, were challenged on day 84 of the experiment (0 dpc) (arrow), corresponding to 84 dpv/63 dpb for group ‘Prime-boost’ and 21 dpv for Group ‘Single’. The average daily body temperature of group ‘Prime-boost’ (open squares), group ‘Single’ (open circles) and group ‘Control’ (filled circles) are presented. The total clinical score per group per day after the BTV8 challenge are presented by open bars (groups ‘Prime-boost’ and ‘Single’) and filled bars (group ‘Control’). Note, no observed clinical signs were indicated as 0.1 for visualization.

Intramuscular vaccination with 2× 1 mL of 10^5^ TCID_50_/mL of DISA8 did not cause local reactions, adverse effects or clinical signs, nor an elevation of the body temperature (not shown). Further, after the BTV8 challenge, both vaccinated groups did not develop elevated body temperatures, but neither did the challenge group ‘Control’ (Figure 2). In contrast, the vaccinated groups did not show the disease after the BTV8 challenge, whereas the group ‘Control’ developed mild clinical signs from 9 to 20 dpc (Figure 2).

The total maximum clinical score was 5 for two consecutive days for the group ‘Control’, which was mainly contributed by one animal with an individual clinical score of 3 on these days. In general, the clinical signs were very minor and related to the upper respiratory tract, like coughing and salivation. All the cattle of group ‘Control’ showed coughing on at least two consecutive days. Apparently, the challenge virus BTV8/net07/e1/bhkp3 was not very virulent in cattle. Obviously, the cattle model is less suitable to study protection against Bluetongue disease.

#### 3.2.2. DISA8 Protects against Viremia and Is Distinguishable by DIVA PCR Testing

**Figure 3:**
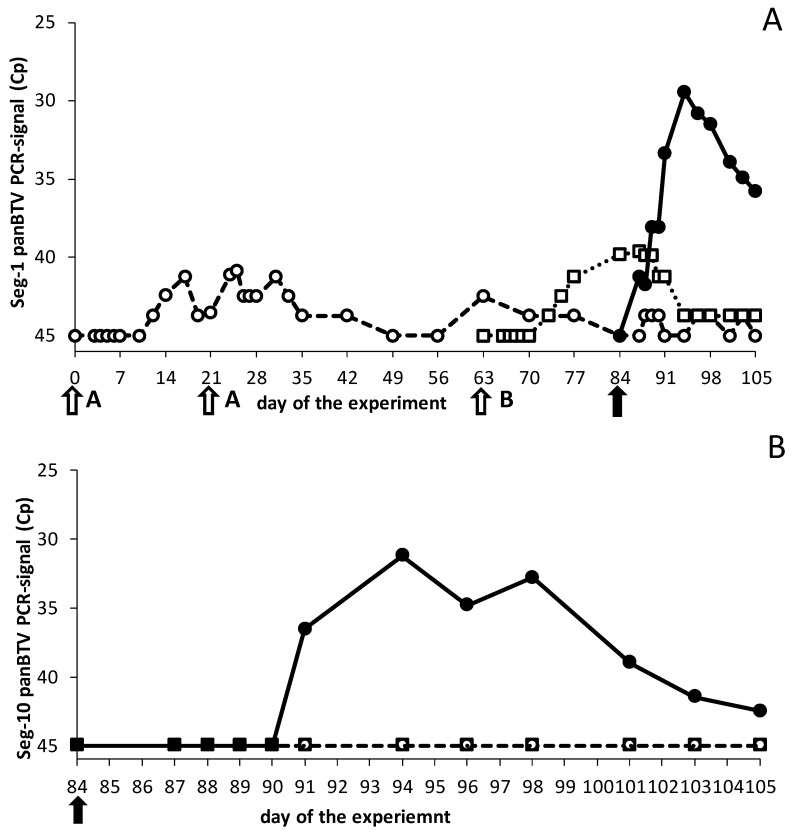
PCR results of the Seg-1 and Seg-10 panBTV PCR tests. (**A**) Average Cp value per group for the Seg-1 panBTV PCR test. (**B**) Average Cp value per group for the Seg-10 panBTV PCR test (DIVA PCR test). Group ‘prime-boost’ (open circles) was vaccinated twice with an interval of three weeks on days 0 and 21 (white arrows). Group ‘single’ (open squares) received one DISA8 vaccination on day 63 (white arrow). Both vaccinated groups, together with unvaccinated group ‘Control’ (filled circles), were challenged on day 84 of the experiment (black arrow). Note, DISA-vaccinated groups were negative for the DIVA PCR test before the BTV8 challenge on day 84 of the experiment (not shown), and the PCR-negative value was set at 45.

The Seg-1 panBTV PCR test detects both the DISA/DIVA vaccine platform and all BTV serotypes, whereas the Seg-10 panBTV PCR test specifically detects BTV8 and not the DISA/DIVA vaccine platform due to the in-frame 72-aa deletion in Seg-10 (Figure 1). Indeed, the vaccinated groups remained negative with the Seg-10 panBTV PCR test up to the BTV8 challenge (not shown). In contrast, very high mean Cp values of >40 were measured with the Seg-1 panBTV PCR test after the first and second vaccinations (Figure 3). Some vaccinated cattle developed Cp values of >38 (not shown). After the BTV8 challenge, most of the cattle of group ‘Single’ remained PCR-negative by Seg-1 panBTV PCR testing, while group ‘Prime-boost’ showed an increasing mean Cp value (Figure 3). These results indicated that the BTV8 challenge did not result in viremia in DISA8-vaccinated cattle. Indeed, the results with the DIVA PCR test were all negative, confirming a lack of BTV8 replication (Figure 3). In contrast, all animals of group ‘Control’ showed obvious viremia by both PCR tests, resulting in mean Cp values of 25–30 for several days post the BTV8 challenge (Figure 3). Taken together, viremia by virulent BTV8 was completely blocked in both vaccinated groups at three weeks post the single vaccination and at nine weeks after the boost vaccination, respectively. We concluded that DISA8 induced an early and lasting sterile immunity in cattle.

#### 3.2.3. The Immune Response against DISA8 Is Serotype Specific and Distinguishable

**Figure 4:**
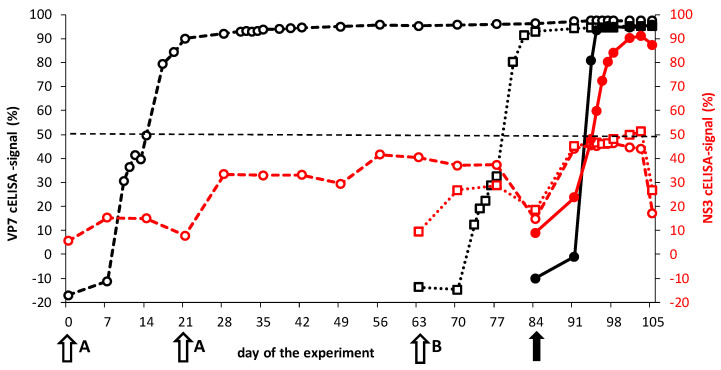
ELISA results of VP7 and NS3 cELISAs. Group ‘Prime-boost’ was vaccinated twice with an interval of three weeks on days 0 and 21 (white arrows indicated **A**). Group ‘Single’ received one DISA8 vaccination on 0 dpv, corresponding to day 63 of the experiment (white arrow indicated **B**). Both vaccinated groups, together with group ‘Control’, were challenged on day 84 of the experiment 0 dpc) (black arrow). Average blocking % per group for the VP7 cELISA and for the experimental NS3 cELISA are in black and red, respectively. Group ‘Prime-boost’ (open circles), group ‘Single’ (open squares) and group ‘Control’ (filled circles).

First, the Ab response was examined by the VP7 cELISA (Figure 4). Vaccinated groups ‘Prime-boost’ and ‘Single’ seroconverted equally to >50% blocking in the third week after the first DISA8 vaccination. VP7 seroconversion reached 95–100% blocking at 21 dpv, corresponding to the day of the boost vaccination for group ‘Prime-boost’ and the day of the challenge for group ‘Single’ (Figure 4). After the boost vaccination of group ‘Prime-boost’, a further increase of VP7 Abs could not be measured by standard procedures with the VP7 cELISA. However, VP7 Abs maintained 95–100% blocking for nine weeks, up to the day of challenge at 84 dpv/63 dpb (Figure 4). Therefore, no further increase of VP7 Abs could be observed after the BTV8 challenge. As expected, group ‘Control’ quickly seroconverted (>50% blocking) in the second week after the BTV8 challenge. Clearly, the raise of VP7 Abs by the BTV8 infection was faster than after the DISA8 vaccination.

The previously developed experimental NS3 cELISA was used to determine NS3 Abs [39]. This cELISA uses coated NS3del containing the immunogenic 72-aa region, which is not expressed by DISA8. Therefore, we suggested that this NS3 cELISA could be an accompanying DIVA test of DISA8 (Figure 1). Group ‘Prime-boost’ and B showed blocking percentages of <30% after one DISA8 vaccination; however, the boost vaccination of group ‘Prime-boost’ showed an increased mean blocking percentage of 40% (Figure 4). One animal of group ‘Prime-boost’ showed >50% blocking on several days post the boost vaccination (not shown). Group ‘Control’ seroconverted rapidly to >50% blocking at 10 dpc and to 80–95% blocking in the third week post the BTV8 challenge. By increasing the cut-off to 50% blocking, the DISA8-vaccinated groups ‘Prime-boost’ and ‘Single’ can be differentiated from the BTV8-infected group ‘Control’, although not all individual vaccinated cattle were interpreted as negative for NS3 Abs prior to the BTV8 challenge. The BTV8 infection of the DISA8-vaccinated cattle also resulted in increased NS3 Abs. Further, two out of four cattle of each vaccinated group seroconverted to >50% blocking after the BTV8 challenge (not shown). Still, the mean blocking % was lower than for group ‘Control’ (Figure 4). Apparently, NS3/NS3a expression after the BTV8 challenge of the DISA8-vaccinated cattle was very poor and led to a much weaker NS3 response. These results indicated that BTV8 replication in DISA8-vaccinated cattle was strongly reduced. Altogether, the lower NS3 Ab response in DISA8-vaccinated cattle after the BTV8 challenge compared to naïve cattle infected by BTV8 supported sterile immunity due to the DISA8 vaccination and indicated complete protection.

**Figure 5:**
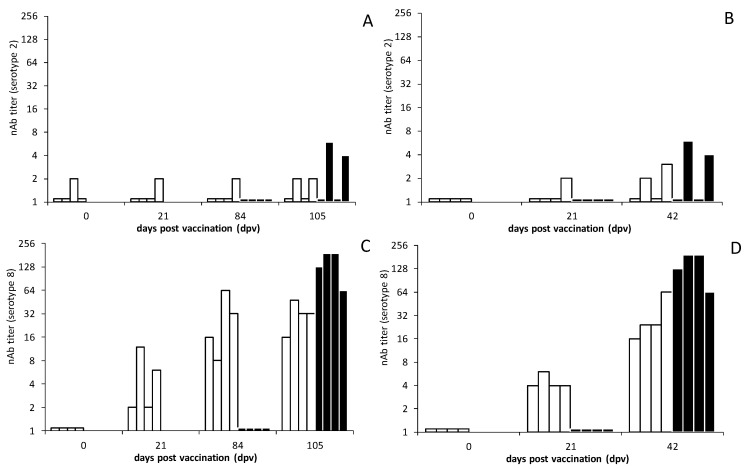
nAb titers by the serum neutralization tests for serotypes 2 and 8. Group ‘Prime-boost’ was vaccinated twice with an interval of three weeks on days 0 and 21 dpv and challenged on 84 dpv ((**A**,**C**), open bars). Group ‘Single’ received one DISA8 vaccination on 0 dpv and was challenged on 21 dpv ((**B**,**D**), open bars). Group ‘Control’ was challenged on the same day (0 dpc), corresponding to day 84 and day 21 for group ‘Prime-boost’ and group ‘Single’, respectively (filled bars). Sera were tested for nAbs against serotype 2 (**A**,**B**) and serotype 8 (**C**,**D**). The highest serum dilution preventing complete CPE formation in the BSR cells is indicated. Note, negative tested sera are indicated.

To determine the titer and serotype specificity of nAbs, SNTs for serotypes 2 and 8 were performed for sera of the indicated days (Figure 5). nAb titers were negative on dpv or 0 dpc. The DISA8 vaccination induced nAb titers against serotype 8 of 2-16 at 21 dpv in both group ‘Prime-boost’ and B. In group ‘Prime-boost’, the nAb titer further increased after a boost vaccination to 8-64 at 84 dpv/63 dpb. Group ‘Prime-boost’ did not develop higher nAb titers against serotype 8 after the BTV8 challenge. The nAb titers varied between 16 to 48 at three weeks post the BTV8 challenge, corresponding to 105 dpv (Figure 5). In contrast, the single-vaccinated group ‘Single’ developed slightly higher nAb titers of 16-64 at three weeks after the BTV8 challenge, corresponding to 42 dpv (Figure 5). Remarkably, the group ‘Control’ showed slightly higher nAb titers of 64-192 at 21 dpc than both vaccinated groups.

The serotype specificity of nAbs raised by DISA8 were determined by SNT with heterologous BTV serotype 2 (Figure 5). The nAb titers were negative at 0 dpv/0 dpc (indicated as 1.1 for clarity), but one animal of group ‘Prime-boost’ showed a very low nAb titer of 2. DISA8 vaccination did not induce nAbs for serotype 2, although a few cattle showed a very low nAb titer, like the one animal on 0 dpv. The DISA8-vaccinated cattle did not develop higher nAb titers for serotype 2 after the BTV8 challenge. Surprisingly, two cattle of group ‘Control’ challenged with BTV8 developed low nAb titers of 4 and 6 against serotype 2 (Figure 5). The differences between the nAb titers for serotypes 2 and 8 confirmed the raising of serotype-specific nAbs and suggested protection against virulent BTV8 but, likely, not against virulent BTV2. We concluded that DISA8 induces a serotype-specific protection in cattle—in particular, after the prime-boost vaccination.

## 4. Discussion

The previously developed DISA/DIVA vaccine platform was based on the LAV-related strain BTV6/net08 lacking NS3/NS3a expression, and a prototype vaccine for serotype 8 has been extensively studied in sheep, reviewed in [29]. We have shown that an in-frame 72-aa deletion accomplished the DISA principle, since the virus release and virus propagation in blood-fed midges were abolished [42]. Here, the 72-aa deletion in LAV was investigated in cattle. The previous BT vaccine platform has been applied for several serotypes by the single exchange of Seg-2[VP2] [41]; however, the exchange of both the outer shell proteins is more flexible than a single Seg-2[VP2] exchange [47,57]. Prototype DISA8 with both outer shell proteins of serotype 8 was generated; as DISA vaccines, for as much as possible, serotypes are foreseen, aiming tailor-made polyvalent DISA vaccines or even broad protection. Finally, the DISA/DIVA vaccine has not been studied in cattle so far, although cattle are considered as the main virus source for the ongoing BTV spread by a high and lasting viremia. Therefore, the prototype DISA8 was studied here in cattle on its safety, DIVA and efficacy and, in particular, on the viremia after the BTV challenge by sensitive PCR diagnostics.

We hypothesized that all features of the multifunctional NS3/NS3a protein are abolished by the 72-aa deletion encompassing Late Domain. A similar in-frame deletion of Late Domain in Seg-10 of AHSV resulted in a promising AHS DISA/DIVA vaccine platform [43,44]. The new BT DISA/DIVA vaccine platform based on the LAV backbone of BTV6/net08 and the 72-aa deletion in Seg-10[NS3/NS3a] was used to generate prototype DISA8 with Seg-2[VP2] and Seg-6[VP6] of serotype 8. DISA8 did not cause clinical signs nor a rise in the body temperature (Figure 2). However, the used LAV backbone of BTV6/net08 is not virulent in cattle [48]. Consequently, the cattle model is less suitable to study the safety of DISA8 and the 72-aa deletion in this backbone in particular. The safety of the BT vaccine candidates with regards to the clinical signs and disease should be studied in susceptible sheep species. More preferably, the 72-aa deletion should be studied in virulent BTV to demonstrate its role in virulence, as previously shown for the NS3/NS3a knockout mutant in virulent BTV8 in sheep [38], and a similar 77-aa deletion in virulent AHSV in ponies [43].

One of the aims of this study was to investigate the vaccine safety in cattle with regards to viremia after the vaccination. The DISA8 vaccination resulted in Cp values of >38 in cattle (Figure 3), whereas the sheep remained PCR-negative in previous studies [38,52,58]. Likely, the replication of DISA8 is higher in cattle and might lead to PCR positivity. The vaccination with a 100-times higher dose of the replicative, abortive BT DISC vaccine also resulted in Cp values of >35 [53], and a similar high antigen load of inactivated BT vaccine could also result in temporary PCR positivity [59,60]. Nevertheless, viremia with Cp values of >38 is expected to be too low for uptake by midges, and the propagation of the DISA vaccine in midges is blocked by the lack of a functional NS3/NS3a protein [33,42]. In conclusion, the new DISA/DIVA vaccine platform with the 72-aa deletion is safe.

For the first time, the efficacy of the DISA vaccine was studied in cattle by intramuscular vaccination. DISA8 induced early, as well as lasting, protection (Figure 2, Figure 3 and Figure 4). Previously, the subcutaneous vaccination of sheep with the prototype DISA vaccine lacking NS3/NS3a with a single Seg-2[VP2] exchange for serotype 8 induced early and serotype-specific lasting protection against disease and viremia [38,52]. Here, sterile immunity was also demonstrated in cattle (Figure 3A,B). While residual DISA8 was still present after the challenge, as detected with the Seg-1 panBTV PCR test, viremia was not measured with the Seg-10-targeting panBTV PCR test or the accompanying DIVA PCR test, which differentially detect wild-type BTVs, including the challenge BTV8. Its protection against other serotypes was not investigated; however, the clear difference between nAb titers for homologous serotype 8 and heterologous serotype 2 suggested serotype-specific protection (Figure 5). These findings are in agreement with previous studies in sheep demonstrating a lack of protection against heterologous virulent BTV2 [52].

The VP7 seroconversion after the prime vaccination with DISA8 (groups ‘Prime-boost’ and B) was remarkably later than after the BTV8 infection (group ‘Control’) (Figure 4). The NS3/NS3a knockout DISA vaccine induced VP7 seroconversion in sheep in the second week, irrespective of the vaccination route [38,52,58]. Similar differences between the VP7 seroconversion in sheep and cattle have been observed for the BT DISC vaccines and BTV6/net08 [47,48,53]. Apparently, cattle seroconvert slightly slower than sheep. Nonetheless, DISA8 completely protected the cattle against viremia as early as three weeks post-vaccination. Due to the low nAb titer for serotype 8, early protection is likely not serotype-specific, as previously shown for ‘serotyped’ BT vaccines, based on BTV6/net08 with exchanged outer shell proteins [50]. Altogether, the vaccination of cattle primarily aims at the reduction of viremia of wild-type BTV in order to diminish the onward spread of more susceptible ruminant species like sheep. We concluded that the DISA/DIVA vaccine platform with 72-aa deletion was highly efficacious and suitable to generate DISA/DIVA vaccines for more serotypes.

Obviously, the deletion of the 72-aa codons in Seg-10 encompassed at least a part of the respective PCR target, causing the differential detection of wild-type BTV (DIVA PCR test) (Figure 1). Several Seg-10 panBTV PCR tests have been developed, reviewed in [54]. Since these PCR tests target the 72-aa region, the DISA/DIVA vaccine platform is DIVA-compatible with all Seg-10 panBTV PCR tests, including the OIE-recommended panBTV PCR test [7,61], similar to the previous NS3 knockout DISA/DIVA vaccine platform [52,54].

Further, the DISA8 vaccination of groups ‘Prime-boost’ and ‘Single’ led to a very weak NS3 Ab response, whereas group ‘Control’ seroconverted rapidly for both VP7 and NS3 Abs (Figure 4). Indeed, the 72-aa region missing in DISA8 encompassed the most immunogenic part of the NS3/NS3a protein from LD to approximately the N-terminus of TM1 [39]. The experimental NS3 cELISA has been used as the DIVA ELISA, accompanying the NS3/NS3a knockout DISA vaccine in sheep and using a cut-off value of 30% blocking [52]. Here, DISA8-vaccinated cattle developed a NS3 Ab response up to a mean blocking of 50% (Figure 4). Even more, several vaccinated cattle showed >50% blocking (not shown). Apparently, the remaining NS3/NS3a deletion protein of DISA8 induced NS3 Abs. However, DISA8 does not express the 72-aa region containing the epitope of the competing monoclonal antibody (MAb) 33H7 of the experimental NS3 cELISA (Figure 1) [39]. We suggest that the remaining NS3/NS3a deletion protein of DISA8 induced NS3 Abs that could hamper the binding of MAb 33H7. Still, the NS3 seroconversion of BTV-infected cattle (group ‘Control’) was very high compared to the DISA8 vaccination, demonstrating that NS3 cELISA could be suitable as the DIVA ELISA, as previously shown for sheep [39,52]. Clearly, the experimental NS3 cELISA should be optimized and validated, since the use of a higher cut-off of 50% than for sheep sera will not prevent the detection of DISA8-vaccinated animals. Alternatively, a smaller NS3del antigen like a 72-aa peptide could be used as a coated antigen to minimize the background by Abs raised by the NS3/NS3a deletion protein of this DISA/DIVA vaccine platform.

## 5. Conclusions

The new DISA/DIVA vaccine platform based on the LAV strain BTV6/net08 with the in-frame deletion in NS3/NS3a Seg-10 and exchange of both the outer shell proteins exhibits an excellent vaccine profile in cattle, which is very similar to the previous platform based on the knockout of NS3/NS3a expression studied in sheep with regards to safety and efficacy, reviewed in [29,31]. The 72-aa deletion accomplished DISA (double blockade of the vaccine spread by midges) and DIVA (by ELISA and PCR tests). However, the serological DIVA should be improved by optimizing the experimental NS3 cELISA or the development of a next-generation NS3 peptide cELISA. Further, the BT DISA/DIVA vaccine platform is suitable to deliver safe, protective DISA vaccines for many serotypes by the exchange of serotype-specific outer shell proteins that can be produced in established vaccine production facilities. Most importantly, these vaccines can be freely and safely combined in polyvalent DISA/DIVA vaccines, aiming for tailor-made or even broad protection to combat multiple serotype situations. Polyvalent DISA/DIVA vaccines of economically important BTV serotypes should be investigated in sheep and cattle.

## Data Availability

Data is contained within the article or Supplementary Materials.

## Supplementary Materials

The following are available online at https://www.mdpi.com/article/10.3390/v13050857/s1, Table S1: Clinical score table.
