# Supplementary material for: The Bluetongue Disabled Infectious Single Animal (DISA) Vaccine Platform Based on Deletion NS3/NS3a Protein Is Safe and Protective in Cattle and Enables DIVA
Source: Viruses. 2021 May 7;13(5):857. doi: 10.3390/v13050857 (PMC8151055; doi:10.3390/v13050857)
Supplement: Supplementary file 1 [file viruses-13-00857-s001.zip › viruses-1184301-supplementary.pdf]

Table S1. CLINICAL SCORE TABLE – BTV INFECTION

	Score 0	Score 1	Score 2	Score 3
**Depression**	Responsive (sheep is alert and responsive to environmental stimuli)	Sheep is listless	Apathy, sheep react only if stimulated, separating from the flock	Prostration, not responsive to stimuli
**Salivation**	No secretion	Lower jaw wet from saliva	Sheep is actively secreting	Hypersalivation
**Facial oedema**	Absence of oedema	Local oedema	Multiple oedemas localized in different regions	Marked facial oedema, “bottle jaw” (submandibular oedema)
**Lower airway distress**	Normal respiratory rate* (≤ 40 breath/min)	Slightly increased respiratory rate* (41-60) and/or abnormal breathing sounds	Increased respiratory rate* (61-100) and/or clear abdominal breathing	Strongly increased respiratory rate* (>100) and/or rapid abdominal breathing and/or mouth breathing/abnormal breathing sound (froth)
**Nasal discharge Upper airway distress**	Absence of ocular/nasal clinical signs	Intermittent serous (clear) to mucoid (gray and cloudy) discharge/lacrimation	Persistent mucoid (gray and cloudy) to purulent (thick and yellowish/green) discharge	Purulent (thick and yellowish/ green) to bloody discharge
	Normal breathing	Rare coughing/sneezing (only during physical activity)	Frequent coughing/sneezing (also at rest)	Frequent coughing/sneezing at rest with prolonged episodes
**Muco-cutaneous and oral lesions** **Ocular discharge**	No lesions	Hyperemia and inflammation labial mucosa/gums, blisters appear on oral mucosa	Petechial and ecchymotic hemorrhages in the oral mucosa/oral erosions	Confluent erosions oral mucosa/ulcers
	Absence of ocular clinical signs	Lacrimation/ red mucous membranes	Discharge/conjunctivitis	Cataractal, white haze, conjunctivitis
**Dermal and hoof coronet lesions**	No lesions/normal derma	Reddening of skin above coronary band, sheep doesn’t show any difficulty of movements	Coronitis, warm lower limbs and hooves (body mass is moved to other legs)	Severe lameness, stiff gait, hunched appearance/sheep may kneel/lying down/crippled
